# Visualizing the Maternal Health Journey for Learning Health Systems: Mixed Methods Combined Experience Approach

**DOI:** 10.2196/82944

**Published:** 2026-02-19

**Authors:** Amanda L Joseph, Bilikis Oladimeji, Helen Monkman, Simon R Minshall, Melissa C Tan, Yuri Quintana

**Affiliations:** 1 School of Health Information Science, University of Victoria, PO Box 1700 STN CSC, Victoria, BC, V8W 2Y2, Canada, 1 250 721 8575; 2 Majestic Global Consulting Inc, Vancouver, BC, Canada; 3 The Society of Participatory Medicine Inc, Boston, MA, United States; 4 SheriBell Global LLC, Pittsfield, MA, United States; 5 University of British Columbia Faculty of Medicine, Vancouver, BC, Canada; 6 Delta Hospital, Fraser Health Authority, Delta, BC, Canada; 7 Homewood Research Institute, Guelph, ON, Canada; 8 Division of Clinical Informatics, Beth Israel Deaconess Medical Center, Boston, MA, United States; 9 Harvard Medical School, Boston, MA, United States

**Keywords:** maternal mortality, patient journey map, journey map, persona, data visualization, health disparities, social determinants of health, Sankey diagram, health care inequities, visualized combined experience diagram, learning health system, systems thinking, systems approach, systems view, human-centered, patient outcomes, maternal health

## Abstract

**Background:**

The United States faces a persistent maternal mortality crisis, with rates far higher than those in other high-income nations. The mortality rate among Black women is more than 3 times that among White women. Traditional data visualizations, such as bar and line charts, often emphasize aggregate outcomes, masking inequities and failing to reflect patient-level experiences.

**Objective:**

This study aimed to address the gaps by taking a systems view and developing a Visualized Combined Experience (VCE) diagram, which is an innovative tool that integrates persona-based storytelling with data visualization to provide a more comprehensive understanding of maternal health outcomes. Specifically, the following research questions were explored: (1) How can the VCE diagram approach be applied to illustrate maternal mortality disparities in the United States? (2) To what extent does this integrated visualization technique reveal connections between individual patient experiences and population-level health outcomes that traditional visualization methods do not? (3) How can the VCE diagram inform a learning health system (LHS)?

**Methods:**

This mixed methods study used publicly available quantitative data from the US Centers for Disease Control and Prevention and adapted qualitative data from the ProPublica award-winning investigative series *“Lost Mothers”* to construct the VCE diagram through a seven-step process involving the following elements: (1) composite persona derived from publicly available narratives, (2) journey map illustrating patient experiences and health system touchpoints, (3) emotive elements of the patient, (4) Sankey diagram of population-level maternal mortality outcomes, (5) “closer look” inset to unmask disparities obscured in aggregate data, (6) evaluation, and (7) data integration.

**Results:**

The VCE diagram revealed critical connections between individual experiences and population-level disparities. When examining mortality rates per 1000 births, Black women had a high rate of 51.2, compared with 16.8 for White women, 14.3 for Hispanic women, and 10.2 for Asian women. The relationship between diagnostic delay and population-level mortality was revealed, with the “closer look” inset demonstrating how disparities can be obscured in aggregate data. The VCE diagram supported a more efficient and empathetic understanding of maternal health outcomes.

**Conclusions:**

The VCE diagram bridges micro-level patient experiences with macro-level population data, holding promise to enhance service evaluation, delivery, and design, and improve health care outcomes. The VCE diagram provides a replicable framework for data visualization that highlights systemic disparities often hidden in aggregate data. Moreover, the availability of structured human experience and service outcome data can provide robust context-specific and situational data to foster a culture of organizational learning and continuous improvement via an LHS. The LHS’s knowledge translation loops provide a conduit to improve patient experiences and reduce morbidity and mortality across populations and health systems. Future work will include usability testing across diverse audiences to assess interpretability and refine applications in LHSs.

## Introduction

### Current Landscape of Maternal Mortality

The United States has the highest maternal mortality rate among high-income countries, with the US Centers for Disease Control and Prevention (CDC) reporting that more than 80% of deaths are considered preventable [1-8]. In their 2023 vital statistics report, the CDC reported a 40% increase in maternal deaths from 861 to 1205 and reported a mortality rate of 69.9 deaths per 100,000 live births for Black women, which was 2.6 times the rate for White women (26.6) and Hispanic women (28.0), with no reported data for Asian women that year [9-11]. Following this, in their 2024 report, the CDC reported that maternal mortality decreased from 32.9 to 22.3 deaths per 100,000 live births; however, mortality differed by race, with 13.2 deaths per 100,000 live births for Asian women, 16.9 for Hispanic women, 19.0 for White women, and 49.5 for Black women [12]. Lastly, in their 2025 report, the CDC reported that the maternal mortality rate per 100,000 live births decreased significantly among White women (14.5), Hispanic women (12.4), and Asian women (10.7), but increased among Black women (50.0) [3]. Though the report stated that the mortality increase in Black women was not statistically significant, it was the only race group that showed an increase from 49.5 (in the previous year) to 50.0 deaths per 100,000 live births in 2023 [3,11,12]. These differences persist across income and education levels, reflecting systemic inequities [8,11,13-16].

### Symptoms, Causes, and Impacts of Maternal Morbidity and Mortality

Accordingly, the five most prominent causes of maternal mortality are as follows: (1) infections, (2) complications from delivery, (3) severe bleeding, (4) hypertension, and (5) unsafe abortion [17-19]. Statistically, cardiovascular diseases and hypertensive disorders of pregnancy (before and after) are more prominent in Black women than in White women (during and after pregnancy) and are the leading cause of death [14,20,21]. As hypertensive disorders of pregnancy (eg, pre-eclampsia and eclampsia) simultaneously affect the fetus and mother, their therapeutic management and pathology are uniquely challenging [22]. Pre-eclampsia (historically referred to as “toxemia of pregnancy” [23]) is a complex multisystem disease, diagnosed by sudden-onset hypertension (>20 weeks of gestation) and at least one other associated complication (eg, proteinuria [protein found in the urine] [24], uteroplacental dysfunction [eg, uterus or placenta dysfunction], and maternal organ dysfunction) [22,25]. When left untreated, pre-eclampsia can rapidly progress, leading to serious and long-lasting complications for both the mother (eg, diabetes, cardiovascular disease, increased risk of stroke, and reduced life expectancy) and the fetus (eg, neurodevelopmental disability, perinatal death, preterm birth, and metabolic or cardiovascular disease), which can result in death [22,25,26]. In pre-eclampsia, the mother’s high blood pressure reduces the blood supply to the fetus, which may result in reduced oxygen and fewer nutrients [27].

Eclampsia, another life-threatening pregnancy disorder, poses the highest risk during the first postpartum week; however, it can occur before, during, or after labor and is frequently characterized by the sudden onset of seizures or coma [27,28]. While pre-eclampsia often precedes it, eclampsia adds complexity, as it can be asymptomatic or show sudden symptoms (eg, visual disturbances, altered mental states, and severe headaches), which require immediate medical intervention to safeguard the mother and fetus [27,28]. Hypertensive disorders of pregnancy, especially pre-eclampsia and eclampsia, are leading causes of maternal mortality and disproportionately affect Black women [14,20-22]. Although generally treatable, these conditions can rapidly progress to severe complications, such as “HELLP syndrome” (ie, hemolysis, elevated liver enzymes, and low platelet count), pulmonary edema, and stroke, with rare but fatal outcomes even in hospital settings [25,28-30]. Our focus on eclampsia reflects its complex trajectory and the systemic delays in recognition and treatment that contribute to preventable deaths.

### Population Health Data and Social Determinants of Health

Data have revealed that Black women are dying from pregnancy-related complications disproportionally compared with other races in the United States [3,9,10,12,31]. Nonmedical factors, such as social determinants of health (SDOH), could be contributing to increased maternal mortality and morbidity across the United States [32-38]. The World Health Organization (WHO) defines SDOH as “the conditions in which people are born, grow, live, work, and age” and contextualizes them as “significant drivers of disease risk and susceptibility within clinical care and public health systems” [19,34,39]. Broadly, SDOH can be categorized into five separate but interrelated domains: (1) economic stability, (2) education access and quality, (3) health care access and quality, (4) neighborhood and built environment (eg, buildings, parks or green spaces, water systems, and energy infrastructure [40]), and (5) social and community context [41]. Despite their importance, SDOH factors are frequently overlooked, unidentified, and poorly documented [35,42,43]. When data are collected, they are often not available in a structured format, making it difficult to aggregate, analyze, and share with other care providers. Until recently, these factors were rarely captured in electronic health records, and thus, their utility in root cause analysis of health disparities was hindered by data quality and availability [42].

### Disparities, Medical Provider Shortages, and Health System Implications

Notably, regardless of income, education level, or socioeconomic status, Black women are 3 times more likely to die from pregnancy-related complications when compared with White women in the United States [8,11,13-16,36,38,44,45]. Furthermore, maternal outcome disparities can reflect broader systemic issues within society and have profound implications for public health. Racial disparities can also exist within and between hospitals, further stratifying the experience of patients and complicating access to quality health care services [5,46]. The most current available data from the CDC, delineating maternal mortality by state, revealed that some US states exhibited higher reported maternal mortality rates than others [37,47,48]. Between the years 2018 and 2021, maternal mortality rates per 100,000 live births were the highest in the following US states: Arkansas (43.5), Mississippi (43.0), Tennessee (41.7), Alabama (41.4), Louisiana (39.0), Kentucky (38.4), Georgia (33.9), and South Carolina (32.7) [37,47,48]. Compounding this problem, the shortage of health care workers is a growing issue not only in the United States but also around the globe [49,50]. The WHO has reported that by 2030, there will be a global shortage of almost 14 million health care professionals (eg, physicians, nurses, and midwives) [51]. Moreover, as difficulties in talent recruitment and retention continue, challenges in meeting “supply” (ie, health care providers) and “demand” (ie, patient volumes) will be exacerbated [49,51,52]. Further, with hospital bed shortages anticipated to reach dangerous thresholds as early as 2032, the situation can become more devastating [53]. US health systems are additionally facing scarcities, specifically in maternity service providers, with 13 obstetrician-gynecologists (OB-GYNs) and only 16 midwife providers per 1000 live births [5,54,55]. Adding complexity, many providers report burnout and job dissatisfaction, with OB-GYNs in particular having the highest litigation rates across specialties [5,50,54,55]. Further, exacerbating the health care crisis, the decommissioning of hospitals in rural and urban centers has been accelerating over the past 30 years, with 136 rural closures between 2010 and 2021 alone [56-59]. The cessation of hospital service operations has unquantifiable consequences for impacted communities, with delays in medical care associated with increased transport times, elevated neighboring hospital and emergency department (ED) patient volumes, and increased morbidity and mortality due to a lack of time-sensitive medical or surgical interventions [56-61]. While this study focuses on the United States, this concerning trend is also prevalent globally and has grave implications for patients and health care providers alike (eg, poor health outcomes, increased mortality rates, hospital closures, medical workforce burnout, moral injury, and compassion fatigue) [49-52].

Data continue to demonstrate the severity of environmental factors that cause risks to human health, and in particular, pregnancy-related mortality and other time-sensitive urgent health care scenarios. Moreover, some researchers posit that a person’s geographic location (eg, city, county, state, and zip code) could be a more powerful indicator of their projected health outcomes than their genetic code [32,33,37]. Others argue that “a person’s biggest risk factor isn’t [their] personal preferences or medical needs...but which door [they walk] through” and infer that inconsistencies in medical service provision and capacity also exist [48]. Thus, disparities can not only be indicative of broader health system failures but also reflect the cumulative impact that SDOH present to the health trajectory of marginalized populations. Thus, participatory and system thinking approaches that foster active collaboration and partnerships between patients and health care stakeholders may have merit in assisting with the root cause analysis of poor patient outcomes [35,36,62,63]. Moreover, participatory models of care delivery by their essence increase communication among health care professionals (eg, physicians, nurses, and caregivers), increase patient empowerment, and improve shared decision-making [64,65]. Additionally, participatory approaches hold promise institutionally to improve not only the effectiveness of clinical handovers but also the health system itself [62,64,65]. Understanding the lived and living experiences of the holistic circle of care (eg, patients, caregivers, nurses, and physicians) and human-centered system perspectives are vital and must be expediently adopted by the broader health care collective [66]. Thus, the adoption of the learning health system (LHS) concept is paramount for targeted and scalable transformational change, and the strategic utilization of human experience data can be a conduit for sustained quality improvement and evaluation [66,67]. As LHSs are systems in which informatics, culture, science, and knowledge translation are aligned for innovation and continuous improvement, their benefits lie in their seamless capacity to embed best practices and human experiences into the care process [18,66-69]. By definition, LHSs present opportunities to align multiple stakeholders, integrated systems, human experiences, real-world data, and external evidence through the application of knowledge [18,66-68,70,71]. As such, human-centered contextually driven data, as experienced by patients, providers, and health care stakeholders, hold promise to yield rich precision-level data (eg, process improvement, diagnostic, clinical, and operational data), which can inform LHSs to improve care and health care outcomes [66].

### Objectives

This study aimed to address the gaps by adopting a system view and developing a Visualized Combined Experience (VCE) diagram that integrates population-level data with persona-based storytelling to assess the interconnected nuances that can lead to disparities in maternal health care outcomes. Specifically, this study seeks to investigate the following research questions:

1. How can the VCE diagram approach be applied to illustrate maternal mortality disparities in the United States?
2. To what extent does this integrated visualization technique reveal connections between individual patient experiences and population-level health outcomes that traditional visualization methods do not?
3. How can the VCE diagram inform an LHS?

## Methods

### Study Design and Framework

#### Overview

This study is part of a broader investigative series that uses nontraditional data visualization techniques to interpret publicly available population health data, illustrating the human aspects of big data and how the data may be convergently displayed to effectively inform the health care sector [35]. Specifically, this study used a mixed-methods approach [72,73] to create a VCE diagram integrating qualitative patient narratives with quantitative population health data. This methodology was used, as it provides a deeper, more comprehensive understanding of the drivers and facilitators of maternal mortality in the United States than either approach (ie, quantitative and qualitative) could have achieved independently [72,73].

#### Phase 1

Phase 1 of this series began with an inquiry into the trends of COVID-19 [35] mortalities across race groups (eg, Asian, Black, Hispanic, Native Indian, and White) and resulted in the creation of a novel methodology for COVID-19 mortality analysis [35]. As such, the VCE diagram consisting of a persona, an Experience Journey Map, and a Sankey diagram was designed to illustrate the varied patient outcomes and trajectories, using preliminary COVID-19 data from the Centers for Medicare & Medicaid Services in the United States [35,74], and the patient narrative data were derived from lived experiences of African American men in the United States who died from COVID-19 [75].

#### Phase 2

Phase 2 (this study) refined the phase 1 methodology [35] and applied it to a different health care scenario, specifically to determine maternal health outcomes across race groups (eg, Asian, Black, Hispanic, and White) across the United States. Next, the VCE diagram was developed, integrating the persona and Experience Journey Map to provide a holistic perspective.

#### Quantitative Data

Publicly available maternal mortality data were obtained from the CDC National Vital Statistics System’s Vital Statistics Rapid Release (VSRR) dataset, entitled “provisional maternal death counts and rates” [3,31]. The reporting period was monthly, with values for each race group summed to provide a single figure for each member in the respective race categories. After calculating the values, a new data table was created to detail each group’s totals and the proportion of maternal deaths during the aggregate 2023 reporting period. This dataset was selected because it contained the latest official statistics on maternal mortality in the United States (ie, 2023), as published in the National Center for Health Statistics 2025 report [3,31]. Maternal death in the dataset was defined according to the WHO’s classification as follows: “the death of a woman while pregnant or within 42 days of termination of pregnancy, irrespective of the duration and site of pregnancy, from any cause related to or aggravated by the pregnancy or its management, but not from accidental or incidental causes” [76].

#### Qualitative Data

The qualitative data for the persona and Experience Journey Map were synthesized from cases documented from the Goldsmiths Prized ProPublica *“Lost Mothers”* series [7,77]. The *“Lost Mothers”* investigative journalism project was selected as a data source, as it provided detailed narratives of maternal health experiences from all 50 states, Washington DC, and Puerto Rico [7,77,78]. The series explored why the United States, compared with other countries in the developed world, has the highest per capita health care expenditure but the worst maternal outcomes. It also depicted the intimate stories, lived experiences, and health outcomes of Black mothers across US continuums of care [7,77].

### VCE Diagram Development Cycle

#### Overview

**Figure 1.**
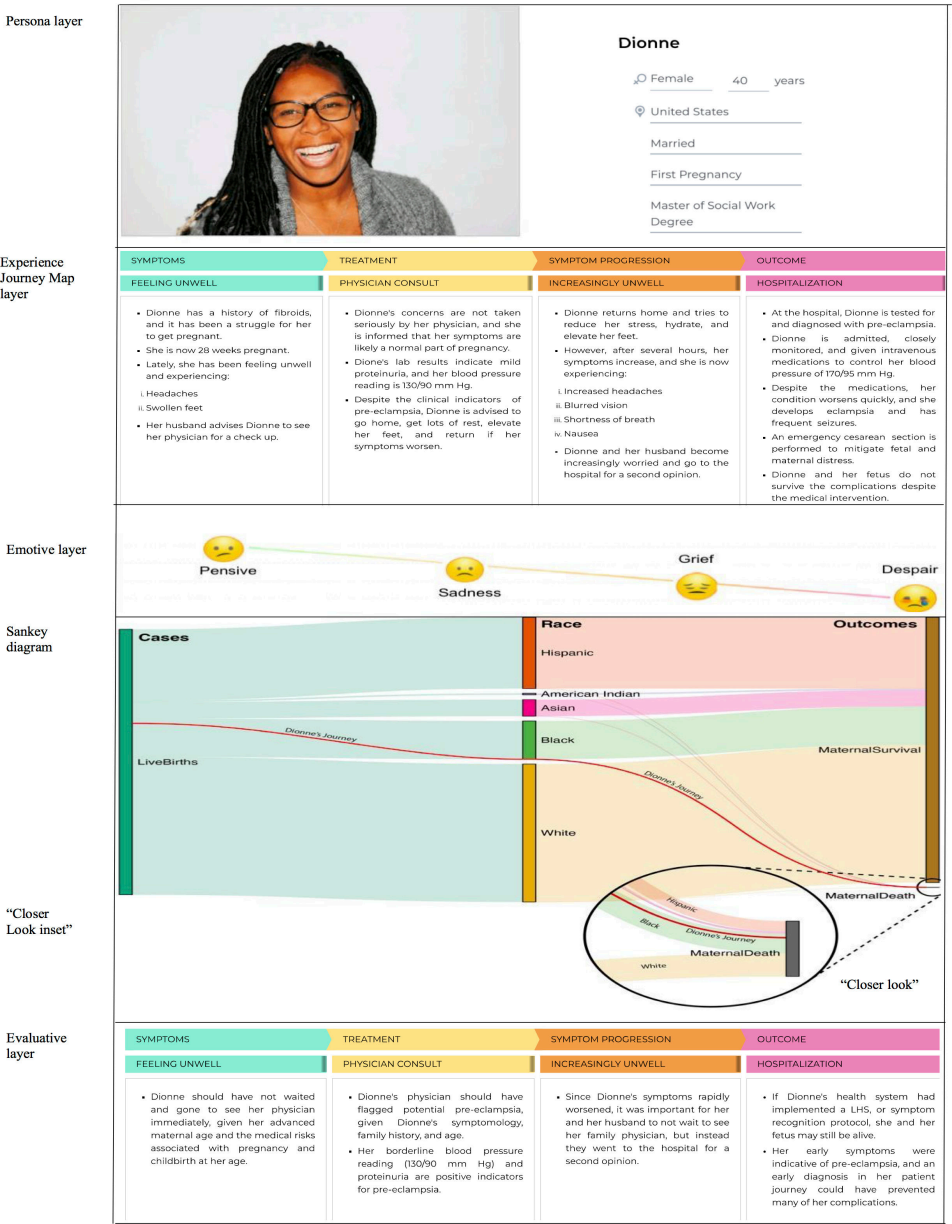
Visualized Combined Experience diagram illustrating Dionne’s trajectory compared with the maternal health outcomes of other population groups. LHS: learning health system.

The VCE diagram has 7 sequential segments (Figure 1), with each corresponding to a layer of the final visualization. The VCE diagram development cycle consists of the following seven steps: (1) persona development, (2) Experience Journey Map development, (3) emotive development, (4) Sankey diagram development, (5) deeper dive - second Sankey diagram development, (6) evaluation, and (7) data integration.

#### Step 1: Persona Development Segment

The persona development segment is the first step of the VCE development cycle. The persona layer is vital as it establishes the contextual background of the analysis and humanizes the numeric health statistics [35,66]. The persona “Dionne” was developed using a structured approach to represent experiences that some Black women in maternal health care settings in the United States may face. The development process included a relational analysis of the narratives and patient experiences depicted in ProPublica’s *“Lost Mothers”* series [7,77]. The analysis identified recurring themes across the maternal health trajectories of Black women; the extraction of demographic information, medical conditions, and health care interactions; and the synthesis of a composite persona incorporating key elements from multiple narratives [7,77]. The persona visualization was developed using a combination of software (UXPressia and Microsoft PowerPoint).

#### Step 2: Experience Journey Map Development Segment

The Experience Journey Map development segment, which is the second layer of the VCE diagram, provides a visual narrative of Dionne’s patient experience as she navigates the care continuum in search of different modalities of care for her symptoms [35,66,79,80]. It was developed to visualize Dionne’s interactions with the health care system across four key phases: (1) symptoms, (2) treatment, (3) symptom progression, and (4) outcomes. Dionne’s actions (ie, what she did, scheduling appointments, and seeking care) were documented in each phase of her journey. Additionally, the touchpoints (ie, interactions with health care providers and systems) and emotional responses (ie, her feelings during each interaction) were also depicted in the schematic. The Experience Journey Map visualization was developed using Adobe Acrobat Reader, UXPressia, Microsoft PowerPoint, and Microsoft Word software.

#### Step 3: Emotive Development Segment

The emotive development segment is the third layer of the VCE diagram. It illustrates Dionne’s emotional spectrum across the four phases of her patient journey: (1) symptoms, (2) treatment, (3) symptom progression, and (4) outcomes. The richness and complexity of Dionne’s emotions are illustrated in the context of each phase of her patient journey experience (ie, the Experience Journey Map) to provide a balanced and intuitive representation of patient emotions. The visualized emotions in this layer were inspired by the sentimental narratives depicted in the ProPublica series [7,77] and created using Adobe Acrobat Reader, UXPressia, Microsoft PowerPoint, and Microsoft Word software solutions.

#### Step 4: Sankey Diagram Development Segment

Sankey diagrams were selected as a visualization tool, as these diagrams provide an intuitive way to represent proportional flows and disparities that are otherwise hidden in aggregate data. Unlike traditional bar or line charts, which often present mortality rates in isolation, Sankey diagrams emphasize the relative magnitude of outcomes between groups through visually weighted flows, making disparities immediately apparent. Studies in health and policy communication suggest that flow-based visualizations can enhance the comprehension of proportional relationships and inequities when accompanied by clear annotations and guiding narratives [81,82]. For policymakers (eg, local and national governments, international bodies, and public agencies), who must often make rapid decisions based on complex data, Sankey diagrams can reduce cognitive load by simultaneously displaying both absolute counts and proportional differences across populations, enabling quicker recognition of systemic inequities. When paired with persona narratives and journey maps, Sankey diagrams serve as a bridge between statistical abstraction and human experience, reinforcing both empathy and data-driven insight. Traditional visualizations, such as stacked bar charts and small multiples, are effective for summarizing quantitative rates across groups [83], but they often strip away the lived experiences that contextualize those disparities. These methods can reveal numeric differences, yet they fail to illustrate how individual patient trajectories connect to population-level inequities, leaving critical drivers of maternal mortality abstract and impersonal. The VCE diagram directly addresses this gap by linking micro-level narratives to macro-level outcomes, aligning with our research questions that seek to both humanize maternal mortality data and expose structural inequities that are otherwise masked.

Both Sankey diagrams were created with a JavaScript library, Data-Driven Documents (D3), using publicly available population data from the VSRR dataset of the National Center for Health Statistics of the CDC, which reported the incidence of maternal death [31,35,84]. Specifically, the Sankey diagrams were populated by the “provisional maternal death counts and rates” dataset, which provided the incidence of maternal death during childbirth (categorized for age and race). Next, the data were filtered by row, race group, and year of death. Within the dataset, the reporting period was monthly, with values (monthly) for each race group summed to provide a single figure for each member in the respective race categories. After calculating the values, a new data table was then created to detail the totals in each group and the proportion of maternal deaths during the aggregate 2023 reporting period. Following this, the aggregate data table was processed through the D3 software visualization library [84] to render the initial Sankey diagram.

#### Step 5: Deeper Dive - Second Sankey Diagram Development Segment

A second Sankey diagram was created, given that the proportion of maternal death was small when compared with the proportional data of maternal survival across all racial populations and the disparities between groups were not visible in the original Sankey diagram. The creation of the second Sankey diagram involved the same diagrammatic curation methods and the same dataset as in step 4. However, maternal death was expressed in a per-thousand ratio as depicted by a magnified inset with flows labeled with race groupings where space permitted. In lieu of demonstrating a second comprehensive diagrammatic representation, a “closer look” inset was embedded within the VCE diagram for esthetics and effective visual impact.

#### Step 6: Evaluative Segment (Patient Choice, Service Delivery Bottlenecks, and Care Gaps)

The evaluative segment, which is the sixth layer of this comprehensive visualization, provides insights into probable situations that could have been improved to avoid patient disposition. It provides a system assessment of the top journey map quandrant through an analysis of the aforementioned 5 layers: persona development, Experience Journey Map development, emotive development, Sankey diagram development, and second Sankey diagram development. This evaluation assesses the various components contributing to Dionne’s health care outcomes and compares her individualistic patient circumstance across the health care interactions in seeking care. The evaluative layer then provides the best practice suggestions for enhancing patient satisfaction, refining service design, and addressing specific patient circumstances, with suggestions toward a more positive human experience and improved health care outcomes for Dionne’s situational narrative and specific patient circumstances.

#### Step 7: Data Integration Segment - Creation of the VCE Diagram

The last step of the VCE development cycle involves the combination of the stratified data elements: (1) persona, (2) Experience Journey Map, (3) emotive layer, (4) Sankey diagram, (5) second Sankey diagram, and (6) evaluation. To illustrate the micro and macro perspectives chronologically, the VCE diagram was created initially with the micro perspective (ie, the persona and journey map), followed by an application of Sankey diagrams to illustrate the macro perspectives. The persona is positioned first to establish context, the journey map is positioned in the middle to illustrate the individual patient experience, and the Sankey diagram is positioned at the bottom to show population-level outcomes. A combination of software tools was used for the final integration and layout of the segments, including Adobe Acrobat Reader, UXPressia, Microsoft PowerPoint, and Microsoft Word. A red line was added to connect Dionne’s journey outcome with the corresponding flow in the Sankey diagram, visually linking the individual experience to population-level data. Annotations were added to highlight key insights and guide the interpretation of the visualization. This design choice was made to personify the statistical data and promote an empathetic understanding of maternal mortality disparities across the United States. The final evaluation layer provides the best practice suggestions from the data and the scenario contexts to improve service delivery and patient outcomes.

### Evaluation Approach

While formal evaluation of the VCE diagram’s effectiveness is planned for phase 3 of this investigative series, a preliminary internal assessment of the visualization was conducted against established information visualization principles [85-87] and health care data visualization best practices [88-90]. This assessment focused on clarity, information density, visual hierarchy, and narrative coherence. We evaluated how effectively the visualization communicated both the individual narrative and population-level disparities and the connections between these 2 levels of analysis.

### Ethical Considerations

This study used publicly available data and published narratives, with no direct involvement of human participants, and thus, it was exempt from ethics review. As an additional safeguard, ethical aspects were considered in representing maternal experiences to ensure a respectful portrayal of maternal mortality cases. Special attention was paid to accurately represent the structural and systemic factors contributing to racial disparities in maternal health outcomes while avoiding potential stereotypes or oversimplifications of complex health care interactions.

## Results

### Overview

This study showed a novel application of the VCE diagram (Figure 1), which exposed hidden disparities across race groups in the United States. Specifically, Dionne’s unique patient journey was illustrated across the maternal health care continuum. By illustrating the human experience, the VCE diagram personified the CDC’s maternal mortality dataset [31], and its application yielded a layered visualization (Figure 1), which integrated individual patient experience with population-level maternal mortality data, revealing three key findings: (1) the presence of significant disparities in maternal mortality rates across racial groups, (2) the presence of critical gaps in care delivery during the pre-eclampsia diagnostic phase, and (3) the masking effect of aggregated data on racial disparities. This study underscores the complexity of aggregating, cleaning, collecting, and interpreting health care data. While the overview presented in the original analysis stratified the data by race group, it did not demonstrate a true categorical representation of race data inequities; thus, the “closer look” secondary analysis was used to clarify the disparities within the dataset (Figure 1).

### Interpreting the Diagrammatic Representation

The completed VCE diagram was designed to be read from top to bottom, allowing readers to understand an individual’s experience before seeing how it relates to broader population patterns. This interpretation should begin at the micro perspectives (persona and journey map) and move toward the macro perspectives (Sankey diagram). The micro perspectives (ie, persona and journey map) provide insights into the individual (ie, Dionne) and her situational factors, health status, and experience across the care continuum. Following this, the macro perspectives (ie, Sankey diagram) layer is presented and used as a macro lens to augment the previous visualization, which incorporates a holistic presentation and positions Dionne’s experience (illustrated by the red line in Figure 1) in relation to the maternal health outcomes of other race groups in the United States. This structure provides a comprehensive view of maternal mortality by connecting individual experiences to population-level outcomes.

### VCE Segment Analysis and Application

#### Overview

The seven segment analysis stages are as follows: (1) persona development, (2) Experience Journey Map development, (3) emotive development, (4) Sankey diagram development, (5) deeper dive - second Sankey diagram development, (6) evaluation, and (7) data integration.

#### Step 1: Persona Development Segment - Introducing Dionne

Dionne is a 40-year-old woman with a Master of Social Work degree who works with disadvantaged children. Dionne is married to a general surgeon and loves children, but has never been able to conceive. The couple’s high levels of education and wealth, and affluent socioeconomic status provide them with superior access to medical care and specialized consultations. Dionne has been diagnosed with fibroids (ie, benign growths found inside the uterus), a uterine condition prevalent in women of reproductive age who are of African descent [7,77,91,92]. The fibroids have caused Dionne painful (often debilitating) menses since menarche [7,77,91,92]. Fibroids, especially if large, can impact fertility, and this is the reason Dionne has been unable to conceive [7,77,91,92]. While Dionne became resigned that she may never become a mother, she was surprised 6 months ago with a positive pregnancy test. Dionne is now in her third trimester (just over 28 weeks of gestation) and has been experiencing frequent headaches and swollen feet. She assumes that these symptoms are a normal part of pregnancy, but her husband has convinced her to schedule a visit with her physician to get the symptoms checked [7,77,93].

#### Step 2: Experience Journey Map Development Segment - Dionne’s Journey

Dionne’s complicated journey and individual narrative have been simplified for this study via the Experience Journey Map across four phases: (1) symptoms, (2) treatment, (3) symptom progression, and (4) outcomes (Figure 1). Collectively, the 4 phases provide an illustrative application of the experiences that Dionne goes through across her patient journey in seeking medical care to alleviate her symptoms and safeguard her pregnancy.

##### Symptoms: Feeling Unwell

The micro perspectives provide insights into Dionne’s patient experience [7,91-93]. At about 28 weeks of gestation, she has recurring headaches, slight nausea, and progressive pedal edema, which should raise suspicion for pre-eclampsia [22,25,77,93-95]. However, this is Dionne’s first pregnancy, and she assumes that these are just “normal” symptoms and is reluctant to see her family physician. After much apprehension and discussion with her husband, she eventually calls her physician’s office to book an appointment. During the call, the medical office assistant (MOA) informs Dionne that she will need to go to her local laboratory and provide a urine sample for analysis. The MOA reiterates that this must be done prior to her appointment with her physician and informs Dionne that she will email her requisition forms later that day, which she can present at the laboratory. Dionne checks her email the next day, prints the forms from her home computer, and goes to the laboratory.

##### Treatment: Physician Consultation

Dionne mentions her symptoms, concerns, and assumptions to her physician, who validates her assumption that her swollen feet are likely a “normal” part of pregnancy. Her physician also checks her blood pressure and reviews her urinalysis results. Dionne’s blood pressure reading of 130/90 mm Hg is a borderline blood pressure reading, and her laboratory results demonstrate mild proteinuria. Despite these warning signs for pre-eclampsia, Dionne is advised to go home, manage stress, elevate her feet, get lots of rest, and book another appointment if her symptoms worsen [7,24,77,93].

##### Symptom Progression: Feeling Increasingly Unwell

Dionne returns home, tries to reduce her stress, and elevates her feet. However, after several hours, her symptoms progress, with increasing nausea, blurred vision, and headaches, and she is now becoming increasingly short of breath [14,77,96]. Dionne’s husband is concerned about the intensity of her symptoms and cautions her that their baby’s safety may be at risk. He encourages her to seek a second opinion. Dionne calls her mother, who agrees with her husband’s perspective, and they promptly go to the hospital for a second opinion.

##### Outcomes: Hospitalization

Following a long wait in the hospital’s ED, Dionne’s symptoms get significantly worse. At this point, she is struggling to breathe and feels faint. Eventually, she is seen by the attending physician. Her husband articulates that Dionne has just seen her family physician, but they are concerned that her symptoms are progressing and are worried about her mild proteinuria diagnosis and elevated blood pressure (130/90 mm Hg). The attending physician takes their concerns seriously, given that Dionne’s husband is a physician as well, and immediately checks her blood pressure and tests her for pre-eclampsia. By this time, Dionne’s blood pressure has elevated to 170/95 mm Hg, and the attending physician then officially diagnoses her with pre-eclampsia.

##### Outcomes: Mortality

During her admission, Dionne and her fetus are closely monitored, and she is given intravenous antihypertensives to lower her blood pressure, but there is no success [25,77,93]. Despite medications, Dionne develops eclampsia and has seizures. An emergency cesarean section is performed to mitigate fetal and maternal distress and further complications. However, unfortunately, both Dionne and her fetus do not survive despite the emergency intervention [27,28].

### Step 3: Emotive Layer Development Segment - Dionne’s Emotional Journey

Illustrating Dionne’s emotions during each phase of her patient journey (Figure 1) provides valuable and insightful information, which is often overlooked due to the transactional and time-constrained nature of care delivery. The emotive layer provides context for the feelings associated with each time Dionne interacts with the health system in seeking care. This segment facilitates intuitive visual representation and immediately communicates whether the experience was positive or negative at each touchpoint. As a 40-year-old, first-time mother with a high-risk and unexpected pregnancy, her visualized emotions have included pensiveness, sadness, grief, and despair across the 4 phases of her emotive journey.

#### Symptoms: Feeling Unwell - Pensive

The starting point of Dionne’s emotional journey is established. She is pensive as she and her husband are engaged in deep thought to understand the root cause of her symptoms.

#### Treatment: Physician Consultation - Sadness

Dionne returns home from her medical appointment but feels sad as she does not feel that her concerns were heard or adequately addressed by her physician. Prior to the consult, she had been resting and elevating her feet, but it did not help with the swelling or her headaches. Dionne has a bad gut feeling that something is seriously wrong and worries about the safety of her baby, but she tries to remain calm and trust the medical advice she has just been given.

#### Symptom Progression: Feeling Increasingly Unwell - Grief

After several hours and much anxiety, Dionne’s husband and her mother convince her to go to the hospital for a second opinion. Since her previous physician consultation, Dionne struggles to cognitively process that something could be wrong with the pregnancy, and her feelings, behaviors, emotions, and experiences range from denial and isolation to anger, bargaining, depression, and acceptance [62,97]. Furthermore, Dionne struggles with losing hope that she will survive and has intense anticipatory grief that her baby will die [62,97]. She is very angry that her family physician did not take her concerns seriously and is inconvenienced to have to go to the hospital. Along with her cyclic internal emotive struggle, her symptoms (eg, headaches, shortness of breath, swelling, and blurred vision) are increasing in severity.

#### Outcomes: Hospitalization - Despair

Dionne is diagnosed with pre-eclampsia, which progresses to eclampsia. Between the terrifying seizures, she cries uncontrollably, gripped by agony, fear, and despair, unsure if she and her baby will survive.

### Step 4: Sankey Diagram Development Segment - Dionne’s Journey Compared to the Population

The first population-level Sankey diagram revealed quantitative disparities in maternal mortality rates across racial and ethnic groups. When accounting for the population density of each racial group in the United States (Hispanic, 19.1%; Asian, 6.3%; Black, 13.6%; and White, 75.0%) [98], the maternal mortality rates per 100,000 births were as follows: Hispanic women, 14.3; American Indian women, 45.3; Asian women, 10.2; Black women, 51.2; and White women, 16.8 [31]. These rates demonstrated that Black women had the highest maternal mortality rate among all race groups analyzed [31].

### Step 5: Deeper Dive

A secondary analysis was conducted, and an additional Sanky diagram (ie, “closer look” inset) was created to underscore the complexity of aggregating, cleaning, collecting, and interpreting data [90,99]. While the overview presented in the original Sankey diagram analysis stratified the data by racial group, it did not demonstrate a true categorical representation of the racial data inequities. As such, the “closer look” inset revealed that in the aggregate view of the initial Sankey diagram, racial disparities were visually obscured owing to the small proportion of maternal deaths relative to the total number of births. When visualized at an appropriate scale (per 1000 births), the magnitude of disparity became clearly apparent, providing methodological insights into how conventional data display can mask significant disparities.

### Step 6: Evaluative Segment (Patient Choice, Service Delivery Bottlenecks, and Care Gaps)

As demonstrated in Figure 1, we provide hypothetical solutions and probable action paths that Dionne could have taken to potentially improve her health care outcomes across the following four key phases: (1) symptoms, (2) treatment, (3) symptom progression, and (4) outcomes. In the symptom phase, Dionne should not have waited and should have consulted her physician immediately, given her advanced maternal age, her pre-existing conditions, and the medical risks associated with childbirth at her age. Next, in the treatment phase, Dionne’s physician should have flagged potential pre-eclampsia, given Dionne’s symptomology, family history, and age. Moreover, her borderline blood pressure reading (130/90 mm Hg) and proteinuria could be considered positive indicators for pre-eclampsia and should have been further investigated. Following this, in the symptom progression phase, since Dionne’s symptoms rapidly worsened, it was prudent for her and her husband to not wait to see her physician and instead seek immediate medical care at the hospital. Lastly, in the outcome phase, Dionne’s health system should have implemented a symptom recognition protocol and ultimately an LHS, which might have saved her and her fetus. Her early symptoms were indicative of pre-eclampsia, and an early diagnosis in her patient journey could have prevented many of her complications.

### Step 7: Data Integration Segment - Dionne’s Holistic Journey

The integrated VCE diagram demonstrated connections between individual experience and population-level outcomes that traditional visualization methods typically do not account for. The red trajectory line (Figure 1) connecting Dionne’s journey outcome to the corresponding flow in the Sankey diagram showed that her experience represented a systemic pattern affecting Black women in the US health care system and that this was not an isolated case. The combined visualization identified specific clinical decision points (ie, dismissal of early pre-eclampsia symptoms and delayed diagnosis) that could contribute to the population-level disparity observed in the Sankey diagram layer. Most notably, the VCE diagram revealed that individual-level factors (eg, high socioeconomic status, education, and health care access) did not mitigate the disparity observed at the population level, suggesting that SDOH alone cannot explain the disparities in maternal mortality.

## Discussion

### Principal Findings

The VCE diagram (Figure 1) combines multiple perspectives and data points into a cohesive visualization and efficiently illustrates stratified maternal outcomes within the United States. This study used data [31] from the CDC to illustrate the trends in maternal mortality across the US population. Maternal mortality is unacceptably high across all race groups; however, when considering the population density of each group, disproportions exist. Regarding the first research question (“How can the VCE diagram approach be applied to illustrate maternal mortality disparities in the United States?”), it was revealed that the VCE visualization facilitates a holistic assessment of relevant health care stakeholders involved in Dionne’s journey and outlines the gaps in care delivery and the pain points she experienced when seeking health care services. Regarding the second research question (“To what extent does this integrated visualization technique reveal connections between individual patient experiences and population-level health outcomes that traditional visualization methods do not?”), it was revealed that this innovative methodological approach provides a more nuanced and comprehensive understanding of the complex, intersecting factors contributing to maternal mortality disparities. By constructing a multidimensional visual representation that simultaneously contextualizes and maps an individual patient’s health care journey in parallel with population-level demographic outcomes, the VCE diagram highlights systemic health care challenges that conventional visualization methods systematically overlook. Unlike standard epidemiological charts that typically present aggregated data through decontextualized line graphs or bar charts, the VCE diagram bridges the epistemological divide between statistical abstraction and human narrative. Similarly, while patient journey maps have emerged as a valuable tool in service design, they are underutilized in health care and frequently lack a substantive connection to broader population-level health trends [35,66,67,79,80]. The proposed approach innovatively integrates individual patient trajectories with demographic mortality patterns through a sophisticated Sankey diagrammatic representation. The VCE diagram offers a holistic analytical framework that transcends traditional disciplinary boundaries by synthesizing multilayered data visualization techniques. This approach not only renders visible the often invisible mechanisms of health care disparities but also provides a powerful methodological tool for understanding the complex interactions between individual experiences and systemic health inequities. The study also revealed many unique features, including but not limited to implications of bias and SDOH, streamlining health care service delivery, and implications for the health care workforce, including policymakers, health systems, advocacy groups, and researchers.

### Implications of Bias and SDOH

The VCE diagram provides a methodological bridge between quantitative disparity metrics and the qualitative experiences of care that contribute to these disparities, particularly the documented pattern of symptom dismissal experienced disproportionately by Black women (Figure 1). Our findings underscore that maternal mortality variations between racial and ethnic groups cannot be explained by biological factors alone. Most studies have demonstrated that a significant proportion of maternal morbidity and mortality cases are preventable, making health care equity and efficiency imperative [5,19,32,46,48,77,93,100,101]. Both implicit bias (ie, unconscious mental processes leading to associations and reactions) and explicit bias (ie, conscious beliefs and attitudes) permeate health care systems through institutionalized practices, clinical decision-making, and clinician-to-patient communication [102-105].

Historical context further complicates this issue, as many foundational advances in obstetric and gynecologic procedures are embedded in discriminatory practices [106-111], race‐based medicine, and research practices with a questionable ethical basis [105,107,109,112-116]. Medical textbook codification and approaches, such as the Caldwell-Moloy pelvic classification system and the Vaginal Birth After Cesarean (VBAC) calculator, continue to influence gynecological practice despite evidence that obstetric pelvises vary in complex ways, which are not effectively captured by artificial typologies [1,117-122]. Moreover, beliefs and practices regarding biological differences in pain perception and blood coagulation across race groups should be re-examined with modern scientific rigor and evaluated by evidence-based human-centered patient outcome research [110,111,123]. Complicating matters, historical biases can also manifest in contemporary disparities, including higher rates of unplanned cesarian births among Black and Hispanic women, with treatment response associations including but not limited to increased risks for hemorrhage, surgical complications, and postpartum infections [2,37,48,118,122,124,125]. To make matters worse, due to many variables and SDOH factors, women with pre-existing mental health issues (eg, anxiety, depression, and post-traumatic stress disorder [126]) from marginalized groups are also vulnerable to receiving poorer care, and consequently, they could experience more distress during childbirth, resulting in worse health care outcomes [127]. Childbirth is a transformative experience for women, not only physically but also psychologically, and their memories of it are indelible [127,128]. The VCE diagram illustrates how these biases translate into clinical encounters where symptoms, such as headaches, visual disturbances, high blood pressure, and edema (clear signs of pre-eclampsia), may unintentionally be dismissed [8,14,44,45,77,96].

### Streamlining Health Care Service Delivery

The VCE approach can help address inconsistencies in medical education and health care service delivery by clearly representing how individual patient journeys connect to population-level data. Black women in the United States face the highest maternal mortality rates across racial groups [8,11,13,15,16,36,38,45,96], underscoring that access to health care alone is insufficient without addressing biases in care delivery. Addressing maternal mortality comprehensively requires acknowledging these disparities as manifestations of structural discrimination, socioeconomic inequities, and inadequate health care infrastructure. High maternal mortality rates are associated with broader health system weaknesses, which also impact other aspects of care, such as chronic disease management, reproductive health services, and preventive care. Furthermore, it involves addressing the underlying SDOH contributing to unequal health outcomes. By addressing these root causes, health care interventions can promote more sustainable, cost-efficient health system practices, where all individuals have the opportunity to thrive with high-quality maternal care, regardless of race, socioeconomic status, or geographic location. Thus, addressing maternal mortality comprehensively is not just a health priority but also a social imperative. The VCE diagram demonstrates that even for women with optimal socioeconomic factors, racial disparities persist, suggesting that structural racism and bias in clinical encounters play significant roles. By visualizing these connections, the VCE diagram gives stakeholders a more comprehensive understanding of intervention points beyond individual clinical encounters.

### Implications for the Health Care Workforce

#### Overview

The VCE diagram offers significant benefits that extend beyond enhancing public understanding of maternal mortality data. By presenting data in a visually intuitive format, the VCE diagram serves as a powerful tool for various stakeholders who play critical roles in addressing health care outcomes, such as policymakers, health systems, health care providers and administrators, advocacy groups, and researchers.

#### Policymakers

For policymakers, the VCE diagram provides a compelling human-centered visual representation of complex data that traditional presentations (eg, statistical tables and text-heavy reports) often fail to convey succinctly or effectively. The combination of personas, journey maps, and Sankey diagrams visually dramatizes inequalities within the health care system, offering an accessible narrative that can drive policy change and identify organizational barriers to care [35,66]. By simplifying the interpretation of data, the VCE diagram enables policymakers to quickly grasp the scope and scale of maternal mortality issues across different demographic groups. This immediate understanding is crucial for effective decision-making and resource allocation. The holistic visualization (Figure 1) provides a clear delineation of health care disparities that can inform targeted interventions and quality improvement initiatives. Moreover, the VCE diagram could serve as a vital tool in legislative advocacy, helping to rally support for policies aimed at reducing maternal mortality and improving health care outcomes.

#### Health Systems

The rapid pace of technological advancement further complicates health systems and their ability to effectively utilize, interpret, and codify population-level data. The consistent influx of new diversified health information systems and health information technologies, along with their data, often hinders their utility and effectiveness in health systems. Technological heterogeneity often perpetuates institutional data siloes across health systems, with interoperability not being possible. This issue is further complicated by the mechanisms by which health care data are collected, synthesized, and aggregated, inhibiting scalability and comparability of macro-level analysis [35,129]. Health systems struggle to not only remain current with evolving medical research and clinical practices but also manage and process the staggering variety and volume of health care data [90,129]. To combat these challenges, health systems could implement regular reviews of maternal care journeys using the VCE diagram methodology to identify critical intervention points, particularly focusing on the transition between prenatal visits and acute care for conditions like pre-eclampsia.

#### Health Care Providers and Administrators

Health care providers and administrators (eg, physicians, nurses, midwives, doulas, and health care executives) can utilize the VCE diagram as an educational tool to enhance their awareness and understanding of health care outcomes across patient populations. By visualizing patient experiences across different metrics (ie, race, age, and location), providers can recognize patterns of care that may unintentionally perpetuate disparities. Integrating the VCE approach into professional medical training curricula and continuing medical education could propagate the importance of contextual understanding in health care delivery, leading to more empathetic, human-centered care [62,66].

#### Advocacy Groups

Advocacy groups could leverage the VCE diagram as a persuasive communication tool by illustrating personal stories alongside systemic patterns of inequality. The stratified visualization provides the micro-level context of an individual’s circumstances and emotional well-being throughout their health care journey. The macro-level health care outcomes create powerful narratives that contextualize population health data. The combined visual narratives (Figure 1) could serve as a potent tool to emotionally engage audiences, mobilize communities, and influence health care reform. Beyond advocacy, the information gleaned from the VCE diagram can also be applied in different contexts to facilitate information exchange and public education to build skills and create awareness. By making complex health data visible and easy to understand, individuals are better equipped to lead local initiatives. Moreover, this transparency can allow communities to take accountability for their own well-being as independent data stewards and advocates.

#### Researchers

For researchers, the VCE diagram offers a novel methodological approach for analyzing and presenting data on maternal health. Integrating qualitative elements, such as personas and journey maps, with quantitative data (ie, stratified mortality rates depicted in the Sankey diagram) allows for a more holistic understanding of maternal mortality. Researchers can use this combined approach to explore correlations between SDOH and maternal outcomes, generating new insights that may not be evident through traditional data analysis or display methods alone. By presenting data in a format that is both accessible and informative, the VCE diagram encourages dialogue and knowledge exchange among researchers, ultimately fostering a more comprehensive approach to understanding and addressing maternal health disparities. This approach facilitates interdisciplinary collaboration by providing a common visual language that bridges gaps among health care, sociology, clinical medicine, and health informatics.

### Applying the VCE Diagram to Inform an LHS

Lastly, regarding the third research question (“How can the VCE diagram inform an LHS?”), it was revealed that the VCE diagram efficiently illustrates the interpretive complexities of working with large publicly available datasets and reveals that data integrity depends on accurate, timely, and structured processes for curation, cleaning, and aggregation [99,130]. Technological advancement has introduced diversified digital and analytic tools that leverage artificial intelligence (AI) to synthesize data with far greater granularity than was previously possible [131]. The integration of AI technologies, such as machine learning, large language models, natural language processing, and neural networks, into health care underscores the need for accurate, unbiased datasets and registries to avoid propagating existing bias gleaned from historic data [103,132,133]. Figure 1 illustrates the criticality of understanding SDOH and their complex relationships with health care outcomes. It has also highlighted the importance of data-informed decision-making, the relevance of accountability in data curation and practices, and the importance of valuing the lived experiences and perspectives of patients and their families [35,131].

LHSs provide a platform to facilitate knowledge creation, translation, and mobilization of the continuous data and analytics gleaned from health care operations, clinical practice, and patient experiences [67,71,134]. This constant influx of new data could catalyze data-informed decision-making, real-time data synthesis for predictive analytics, and patient outcome simulation to foster continuous organizational learning [66,67,71]. Additionally, quality improvement initiatives and targeted interventions could also manifest, which hold promise to improve patient outcomes and reduce morbidity and mortality [66,67,71]. This continuous supply of data could assist in crafting human-centered care delivery [50,66] that meets the needs and goals of health care stakeholders, including but not limited to the following: policymakers, health systems, health care providers and administrators, advocacy groups, researchers, patients, and formal and informal caregivers. Moreover, the VCE diagram (Figure 1) provides six interrelated data streams to inform an LHS: (1) persona, (2) Experience Journey Map, (3) emotive information, (4) Sankey diagram of population data, (5) closer look inset, and (6) evaluation data. Together, these components demonstrate health inequities while challenging popular beliefs, for instance, showing that low socioeconomic status is not necessarily a determinant of mortality in Black women [15,32,38].

Incorporating SDOH data into LHSs could foster improvements to patient engagement, enabling them to have an active role in shared decision-making as stewards of their own health (should they desire to do so), and it would provide more targeted data for care providers as they would have better insights into the social circumstances of their patients [66,135]. Furthermore, integrating SDOH data into LHSs could cultivate personalized and tailored service provision, which could allow health care providers to circumvent biases inherent in maternal care (eg, the Caldwell-Moloy classification and VBAC calculator) [66,135]. Incorporating human-experience data into LHS activities and initiatives holds endless promise to improve health care outcomes, patient and provider satisfaction, and medical intervention efficacy [35,66,67]. The VCE diagram (Figure 1) illustrates multiple data streams in aggregate, and thus, if used in real-world settings, it could assist in targeted medical diagnostics and catalyze holistic and continuous improvement through simultaneous data analysis and synthesis. Moreover, the data gleaned could inform LHSs and service design, increasing health system scalability and efficiency [136].

### Future Work

A key area for future work is formal user testing to evaluate how different audiences, such as the general public, clinicians, and policymakers, interpret the VCE diagram. While the design was assessed against established visualization principles, the extent of its accessibility and usability across diverse stakeholders remains untested. Clarifying the most appropriate target audiences and assessing their engagement with the VCE diagram will be essential to validate its utility and guide its application in practice.

### Limitations

While the Dionne persona illustrates important aspects of the maternal experience, a single persona cannot capture the true diversity of the patient experience. This study was limited by the quality and accuracy of publicly available maternal mortality data from the CDC, and it is acknowledged that data may fluctuate due to reporting issues on death certificates [12]. Additionally, the VCE diagram used static data from a specific period, which may not reflect current maternal mortality ratios, and the quality of comparisons depends on how the CDC data were classified, coded, and sourced. Additionally, while the study focused on pre-eclampsia among Black women, the authors acknowledge that this condition affects women across all racial groups, and this study intended to not only identify maternal health disparities but also demonstrate how aggregate data can be misinterpreted in different contexts.

### Conclusion

This study demonstrates the novel mixed methodological contribution of the VCE diagram as an innovative human-centered method to integrate qualitative patient narratives with quantitative population-level maternal health data. By combining personas, journey maps, and Sankey diagrams, the VCE diagram offers a structured framework that reveals disparities often obscured by conventional visualization methods. While the current work illustrates its potential to enhance the understanding of maternal mortality, further research is needed to evaluate its interpretability among diverse audiences and its effectiveness as a decision-support tool. Future studies will explore how the VCE diagram can be applied to other clinical domains and tested within LHS contexts. In this way, the VCE diagram serves as not only a novel mixed-methods tool but also a foundation for advancing human-centered approaches to presenting health care data and visualizing health care outcomes.

## Abbreviations

AI: artificial intelligence
CDC: Centers for Disease Control and Prevention
ED: emergency department
LHS: learning health system
MOA: medical office assistant
OB-GYN: obstetrician-gynecologist
SDOH: social determinants of health
VBAC: Vaginal Birth After Cesarean
VCE: Visualized Combined Experience
VSRR: Vital Statistics Rapid Release
WHO: World Health Organization
